# Associations of Mid‐to‐Late Life Neighborhood Disadvantage with Cognitive Trajectories and Dementia Outcomes in the Health and Retirement Study

**DOI:** 10.1002/alz70860_101994

**Published:** 2025-12-23

**Authors:** Adrienne R.S. Lee, Gretchen E Bandoli, Lauren L Brown, Linda K. McEvoy, Eyal Oren, Scott C Roesch, Andrea Z. LaCroix

**Affiliations:** ^1^ Center for Health Disparities Research, University of Wisconsin School of Medicine and Public Health, Madison, WI, USA; ^2^ Herbert Wertheim School of Public Health and Human Longevity Science, University of California, San Diego, La Jolla, CA, USA; ^3^ Leonard Davis School of Gerontology, University of Southern California, Los Angeles, CA, USA; ^4^ San Diego State University, San Diego, CA, USA; ^5^ Kaiser Permanente Washington Health Research Institute, Seattle, WA, USA

## Abstract

**Background:**

Neighborhood socioeconomic disadvantage has been associated with worse cognitive outcomes in cross‐sectional studies. We prospectively investigate associations between mid‐to‐late life neighborhood deprivation, cognitive performance trajectories, and dementia outcomes in a U.S.‐based sample of older adults.

**Methods:**

To measure the exposure of neighborhood‐level socioeconomic disadvantage, we applied the Area Deprivation Index (ADI) to midlife residential addresses and operationalized it continuously and dichotomized at the 15% most disadvantaged neighborhoods. Outcomes included immediate/delayed word recall and serial sevens scores from the Health and Retirement Study (1998‐2020) and algorithmically classified dementia (normal, cognitive impairment/no dementia [CIND], or probable dementia) among cognitively intact adults aged ≥50. Generalized linear mixed models were used to analyze cognitive performance trajectories and Cox Proportional Hazard Models were used to analyze time to dementia. Models adjusted for baseline age, gender, race/ethnicity, childhood socioeconomic status, and educational attainment; differences in associations were examined by race/ethnicity and gender.

**Results:**

The sample (*n* = 26,728) was equally distributed across gender (55.1% women) and education (50.8% completed HS/GED diploma), and 30% came from under‐represented racial/ethnic groups. Residing in high versus low disadvantaged neighborhoods in midlife resulted in worse baseline performance and faster decline in immediate (intercept=‐0.20/slope=‐0.009)/delayed (intercept=‐0.20/ slope=‐0.010) word recall and serial sevens scores (intercept=‐0.13/slope=‐0.007) for White/non‐Hispanic and faster decline among Black adults (immediate recall: slope=‐0.012; delayed recall: slope=‐0.012; serial sevens: intercept=‐0.13/slope=‐0.008). Living in high versus low disadvantaged neighborhoods did not influence baseline cognitive performance or trajectories in Hispanic/Latino adults. Only among White/non‐Hispanic adults living in high versus low disadvantaged neighborhoods increased the likelihood of CIND (HR=1.20, 95% CI: 1.14‐1.26) and probable dementia (HR=1.21, 95% CI: 1.09‐1.13). Residing in high versus low disadvantage neighborhoods resulted in lower baseline performance, faster decline, and increased risk of dementia across genders with larger differences in baseline performance and magnitude of dementia risk in men (HR=1.24, 95% CI: 1.17‐1.32).

**Conclusion:**

In a nationally representative population of US older adults with intact cognition, the level level of residential neighborhood disadvantage in mid‐to‐late life impacted cognitive performance trajectories in White/non‐Hispanic and Black adults. Residing in more highly disadvantaged neighborhoods increased risk of dementia outcomes specifically among White/non‐Hispanic adults.

